# Influence of Temperature and Salt Concentration on the Hydrophobic Interactions of Adamantane and Hexane

**DOI:** 10.1021/acs.jpcb.1c09860

**Published:** 2022-01-13

**Authors:** Małgorzata Bogunia, Adam Liwo, Cezary Czaplewski, Joanna Makowska, Artur Giełdoń, Mariusz Makowski

**Affiliations:** Faculty of Chemistry, University of Gdańsk, ul. Wita Stwosza 63, Gdańsk 80-308, Poland

## Abstract

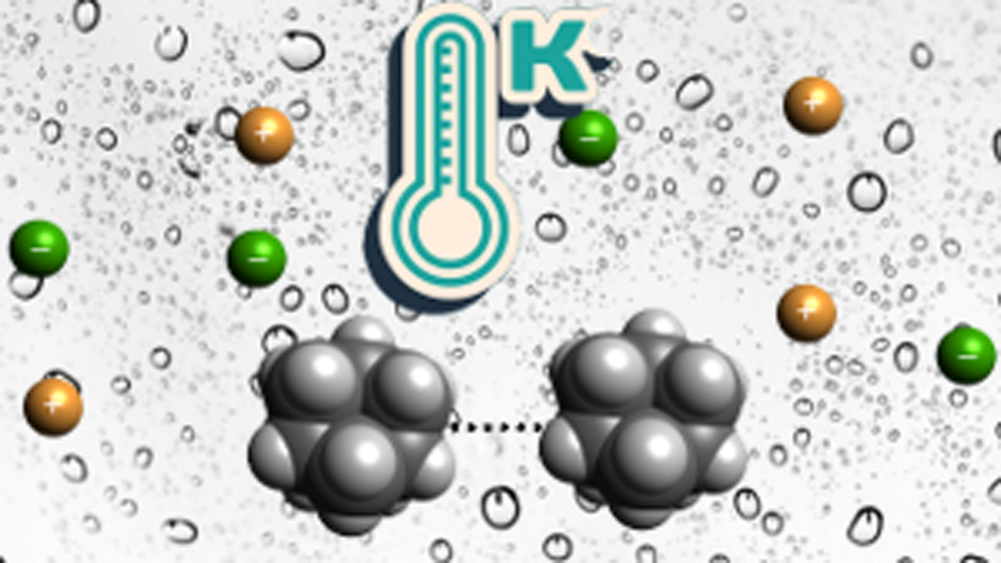

One of the definitions of hydrophobic interactions is the aggregation of nonpolar particles in a polar solvent, such as water. While this phenomenon appears to be very simple, it is crucial for many complex processes, such as protein folding, to take place. In this work, the hydrophobic association of adamantane and hexane at various temperatures and ionic strengths was studied using molecular dynamics simulations with the AMBER 16.0 program and the GAFF force field. The potentials of mean force of hydrophobic dimer formation, as well as the excess free energy, excess energy, excess entropy, and excess heat capacity corresponding to the formation of the contact minimum, were determined and analyzed. For both systems, the depth of the contact minimum in the potential of mean force was found to increase with both temperature and ionic strength. The excess heat capacity of the association at the contact minimum and *T* = 298 K was found to be negative and to decrease, while the excess entropy and energy were found to be positive and to increase for both systems, the changes being more pronounced for the hexane dimer. The excess heat capacity is also greater in absolute value for the hexane dimer.

## Introduction

One of the definitions of hydrophobic interactions is the propensity of nonpolar particles to aggregate in a polar solvent, such as water.^1^ This simple, at the first sight, phenomenon plays a very important role in nature. Hydrophobic interactions are, for example, key driving forces for processes like micelle formation or protein folding.^2^ The essence of the hydrophobic effect could be explained in terms of thermodynamics. The solvation-free energy is lower for aggregated particles than for dispersed ones. Consequently, the solvation-free energy could be considered as the major driving force for the self-assembly of particles.^1,3^ Depending on the size of interacting nonpolar molecules, the differences in the water structure near the solute molecules could be observed. In the case of small particles (with a radius less than 1 nm), hydration leads to the reorganization of water molecules into ordered structures around a solute, which is linked with a hydrogen-bond network. Hydration of bigger molecules (radius above 1 nm) brings broken H-bonds and changes in the water structure. Consequently, the solvation entropy of small hydrophobic molecules is negative and that of larger molecules is positive. In this context, the association of small solutes is entropically driven, while that of larger solutes is driven by enthalpy.^3−5^

The energetics of hydrophobic interactions is often studied by analyzing the potential of mean force (PMF). This quantity primarily depends on the distance between the particle centers and can be interpreted as the free-energy cost of bringing two hydrophobic molecules, immersed in water, from infinitely large separation to a given distance.^6^ The shape of the PMF of hydrophobic interactions typically contains three extrema. The first one, termed the contact minimum (CM), is usually the deepest one and occurs when the two particles are at the closest distance from each other. The second minimum, termed the solvent-separated minimum (SSM), refers to the distance at which one water molecule enters the space between the two monomers. The third extremum is a maximum located between the CM and the SSM and is termed the desolvation maximum (DM). Changes in the depth of the two described minima are directions of changes in the strength of hydrophobic interactions.^6^ Hydrophobic interactions have been studied by integral equation theory. From this theory, Pratt and Chandler^7,8^ derived an expression for the interactions of two spherical solutes in water. It has been affirmed that this expression is independent of solute–solute and solute–solvent interaction parameters. This theory has also been applied to determine entropic and enthalpic contributions to the PMF of hydrophobic molecule association. Based on the integral equation theory and other methods, it has been shown that the CM is stabilized by the entropic term, while the entropic term plays the primary role in SSM stabilization.^6−9^ Parui and Jana,^6^ during their studies for hydrophobes at different temperatures, found a second solvent-separated minimum (SSSM) in the PMF of methane dimers. This minimum is more stable at lower temperatures. The authors concluded that the SSSM in the PMFs of hydrophobic pairs consisting of cyclobutene and a rodlike hydrophobe is more stable than the first SSM at *T* = 240 K. It was also hypothesized that the stabilization of the SSSM could play an important role in the cold denaturation of proteins.^6^

Experimentally, the hydrophobic interactions could be examined by direct measurements of the force acting between hydrophobic surfaces, by using methods such as surface force apparatus (SFA) measurement, atomic force microscopy (AFM), or AFM droplet/bubble probe technique. However, molecular dynamics (MD) simulations remain one of the most valuable techniques for studying hydrophobicity, as it is not possible to directly measure the PMF of pairs of small nonpolar molecules in water.^10,11^

The hydrophobic interactions strongly depend on external conditions of which temperature and salt presence require particular attention because they are critical for the processes that occur in soft matters, especially the biological phenomena. Lüdemann et al. studied the temperature impact on hydrophobic interactions. A stronger association of methane molecules at higher temperatures was shown. The most significant changes occurred at the CM in the temperature range from 300 to 350 K. The solvent-separated minima were barely temperature-sensitive. It was noted that the depth of the CM increases with increasing temperature.^12,13^

Masterton and Lee^14^ reported in 1970 that change of the free energy for cavity formation is more positive in salty water than in pure one. This means that it is harder to create a cavity in the presence of salts. It was also concluded that for small particles, all studied salting coefficients were in quite good agreement with experimental results, but for greater molecules, it was relatively poor.

De Visscher^15^ observed that salting-out is an effect of interactions between ions and water molecules, solutes are not involved as it could be expected. In this case, we can conclude that the water model used in studies could be crucial for obtaining results. The choice of the water model in this paper was suggested by the results of previous studies, which showed that TIP3P results in *T* = 298 K are quite similar to the TIP4PEW model.^17^

Jorgensen et al.^18^ studied the temperature and sodium chloride impact on the solubility of anthracene in water. It was concluded that NaCl has a salting-out effect on the solubility of the studied compound. Coefficients of this effect did not vary significantly with temperature changes. Thermodynamic parameter analysis has shown that transfer of anthracene to water with salt is thermodynamically unfavorable, because of a decrease in entropy.

The model systems used to investigate hydrophobic interactions by molecular simulations typically comprise either small (e.g., methane) or nanoscale-sized (e.g., fullerene) hydrophobes. Therefore, in this study, we selected adamantane, which is a medium-sized rigid hydrocarbon molecule with a nearly spherical shape, and hexane, which is a medium-sized highly flexible hydrocarbon molecule with a prolate shape. Because the strength of hydrophobic interactions heavily depends on both temperature and ionic strength, while studies in which both factors are considered simultaneously are scarce, we included both of them in this study. Consequently, using umbrella-sampling MD simulations, we determined the PMFs of the dimers of these molecules in water at various temperatures and ionic strengths and, subsequently, determined and analyzed the excess thermodynamic quantities for the temperature and salt concentration ranges analyzed.

## Methods

We used umbrella-sampling MD simulations to determine the PMFs of pairs of hydrophobic solutes. The hydrophobic homodimers of adamantane and hexane molecules were investigated. Each dimer was put in a periodic TIP3P^18^ water box with sides of around 60 Å. MD simulations were conducted in two steps. In the first step, each system was equilibrated under the NPT conditions (constant number of particles, pressure, and temperature) at nine temperatures *T* = 273, 285, 298, 310, 323, 335, 348, 360, and 373 K, *p* = 1 atm for 100 ps. Then, the last configuration obtained in the first step was used as the starting point to the next NVT ensemble simulation (constant number of particles, volume, and temperature) at *T* = 273, 285, 298, 310, 323, 335, 348, 360, and 373 K for 10,000 ps. The integration time step was 2 fs. A 10 Å cutoff was used for all nonbonded interactions, and the electrostatic energy was estimated by using the particle-mesh Ewald summation.^19^ For all dimers, a series of 24 (for adamantane) and 11 (for hexane) windows of 10 ns simulation per window were run. A different harmonic restraint potential (eq 1) enforced on the distance (ξ) between two atoms (one from each particle in the dimer) that are closest to the center of the mass of each of the particles was used for every window.
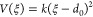
1where *k* is the force constant (*k* = 2 kcal/mol/Å^2^) and *d*_0_ is the equilibrium distance for each dimer (equal to 4.0, 5.0, 6.0, 6.5, 7.0, 7.5, 8.0, 8.5, 9.0, 9.5, 10.0, 10.5, 11.0, 11.5, 12.0, 12.5, 13.0, 13.5, 14.0, 14.5, 15.0, 16.0, 17.0, and 18.0 Å, respectively, for adamantane for windows 1–24, and equal to 4.0, 5.0, 6.0,...,14.0 Å, respectively, for hexane for windows 1–11). The snapshots from MD simulations were saved every 0.2 ps. For each window, 50,000 configurations were generated. The simulations were conducted with 7022 water molecules and a dimer of nonpolar particles. Simulations with ions were also carried out, each system consisting of (1) the respective dimer, 7022 water molecules; (2) the respective dimer, 6922 water molecules, 50 Na^+^, and 50 Cl^–^ ions; (3) dimer, 6778 water molecules, 122 Na^+^, and 122 Cl^–^ ions; (4) dimer, 6662 water molecules, 180 Na^+^, and 180 Cl^–^ ions; and (5) dimer, 6552 water molecules, 235 Na^+^, and 235 Cl^–^ ions. This enabled us to study the influence of ionic strength on hydrophobic interactions at different temperature conditions. The ionic strength values were equal to 0.00, 0.40, 1, 1.5, and 2 mol/dm^3^. In our calculations, we assumed zero charges on the solute atoms. The aliphatic carbon (sp^3^) and aliphatic hydrogen atoms were assigned the CT and HC AMBER atom types, respectively. To determine the PMF, the results from each window were processed by using the weighted histogram analysis method (WHAM).^20−22^ One-dimensional histograms were plotted in the distance between the geometric centers of interacting molecules, the orientations of the solute molecules being averaged out. The calculated PMFs should tend to zero with the increasing distance (after subtracting the constant factor accounting for the hydrophobic hydration free energy of the isolated solute molecule). The PMF baseline value was computed as the average of the PMF distance in the range of 14–15.4 Å for adamantane and 12–13.5 Å for hexane, respectively. The procedure of histogram determination followed that of our earlier work.^16,22−24^

The depth of PMF at the contact minimum distance corresponds to the excess free energy Δ*F*(CM) values associated with the contact minimum formation of each dimer. To obtain reliable estimates of the excess energy, Δ*U*(CM), excess entropy, Δ*S*(CM), and excess heat capacity, Δ*C*_V_(CM), we fitted a quadratic function (eq 2) to the temperature dependence of Δ*F*(CM) (for all nine simulation temperatures, i.e., 273, 285, 298, 310, 323, 335, 348, 360, and 373 K) and, subsequently, determined the quantities mentioned above from eqs 3−5.
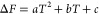
2
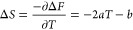
3
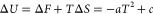
4
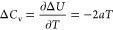
5

## Results and Discussion

### Potentials of Mean Force

**Figure 1:**
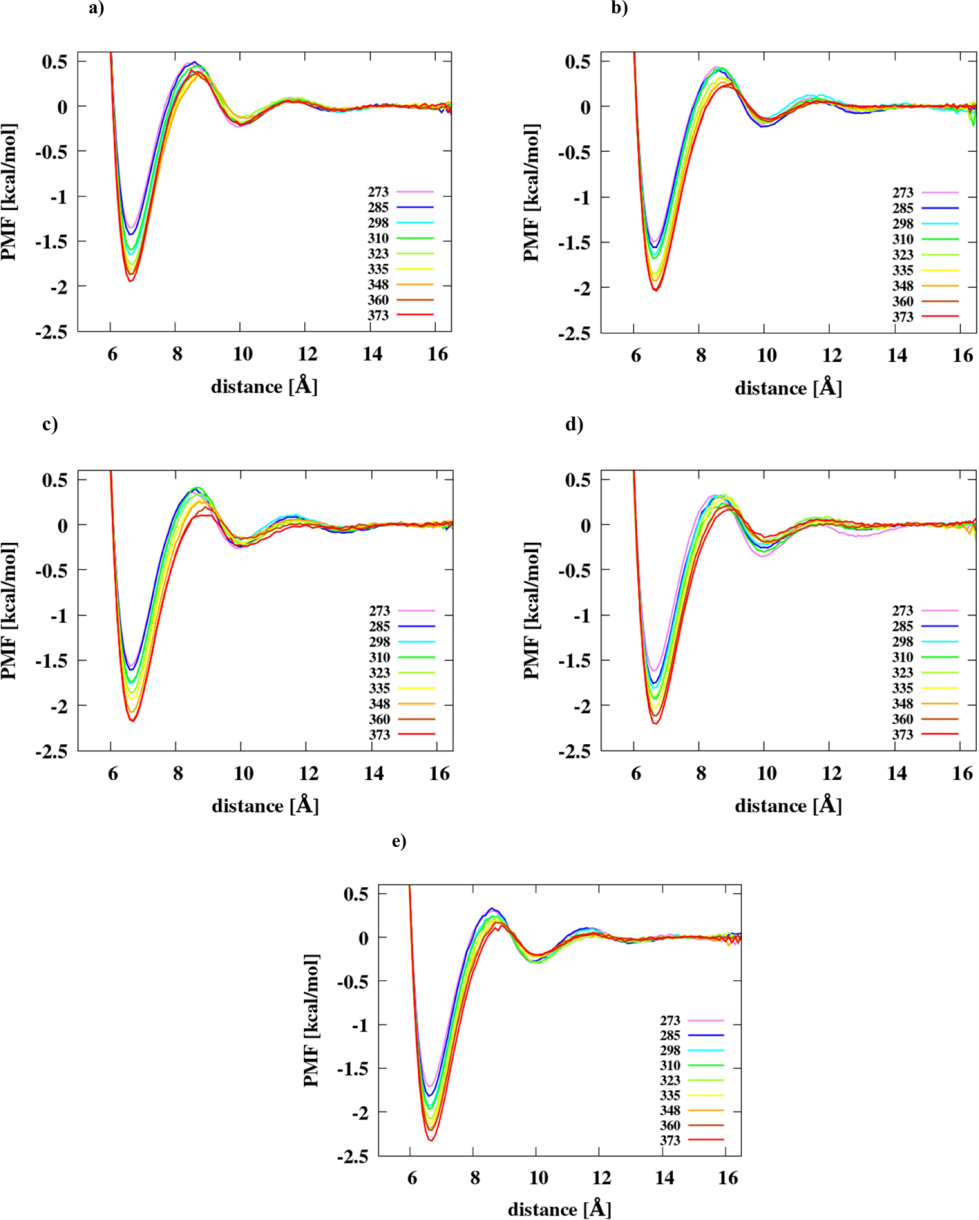
PMFs of the adamantane dimer in (a) IS = 0 mol/dm^3^, (b) IS = 0.40 mol/dm^3^, (c) IS = 1 mol/dm^3^, (d) IS = 1.5 mol/dm^3^, and (e) IS = 2 mol/dm^3^ in the TIP3P water model at different temperatures.

**Figure 2:**
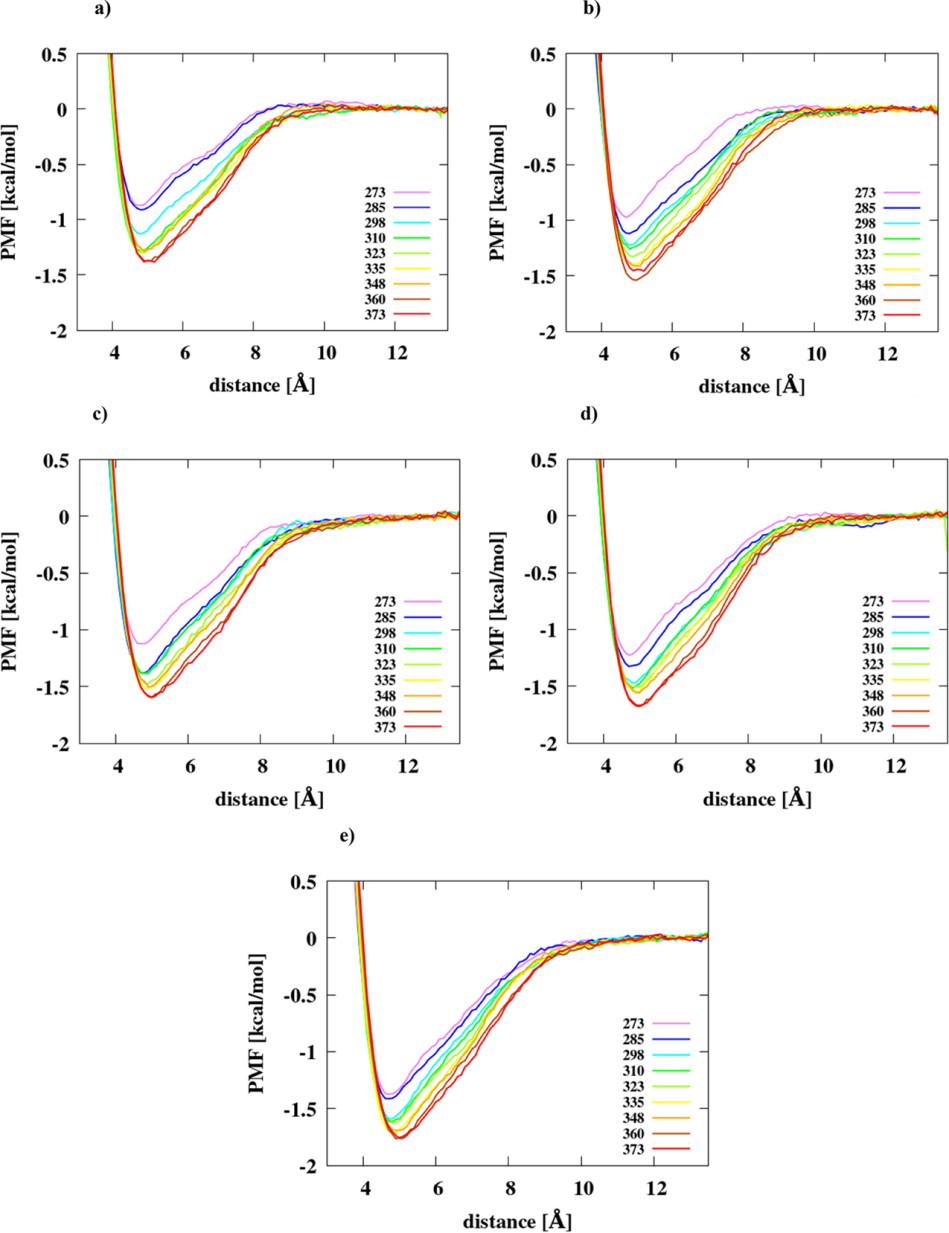
PMFs of the hexane dimer in (a) IS = 0 mol/dm^3^, (b) IS = 0.40 mol/dm^3^, (c) IS = 1 mol/dm^3^, (d) IS = 1.5 mol/dm^3^, and (e) IS = 2 mol/dm^3^ in the TIP3P water model at different temperatures.

The PMF plots in the distance between the mass centers of the hydrophobic particles at different ionic strength values at nine temperatures (273, 285, 298, 310, 323, 335, 348, 360, and 373 K) and ionic strength (0, 0.4, 1.0, 1.5, and 2.0 mol/dm^3^) are plotted as shown in Figures 1 and 2 for adamantane and hexane dimers, respectively, panels A–E of each figure correspond to a different ionic strength value. The PMFs of adamantane exhibit the CM, SSM, and DM. However, for each temperature and ionic strength value, only the CM is observed in the PMFs of hexane.

**Figure 3:**
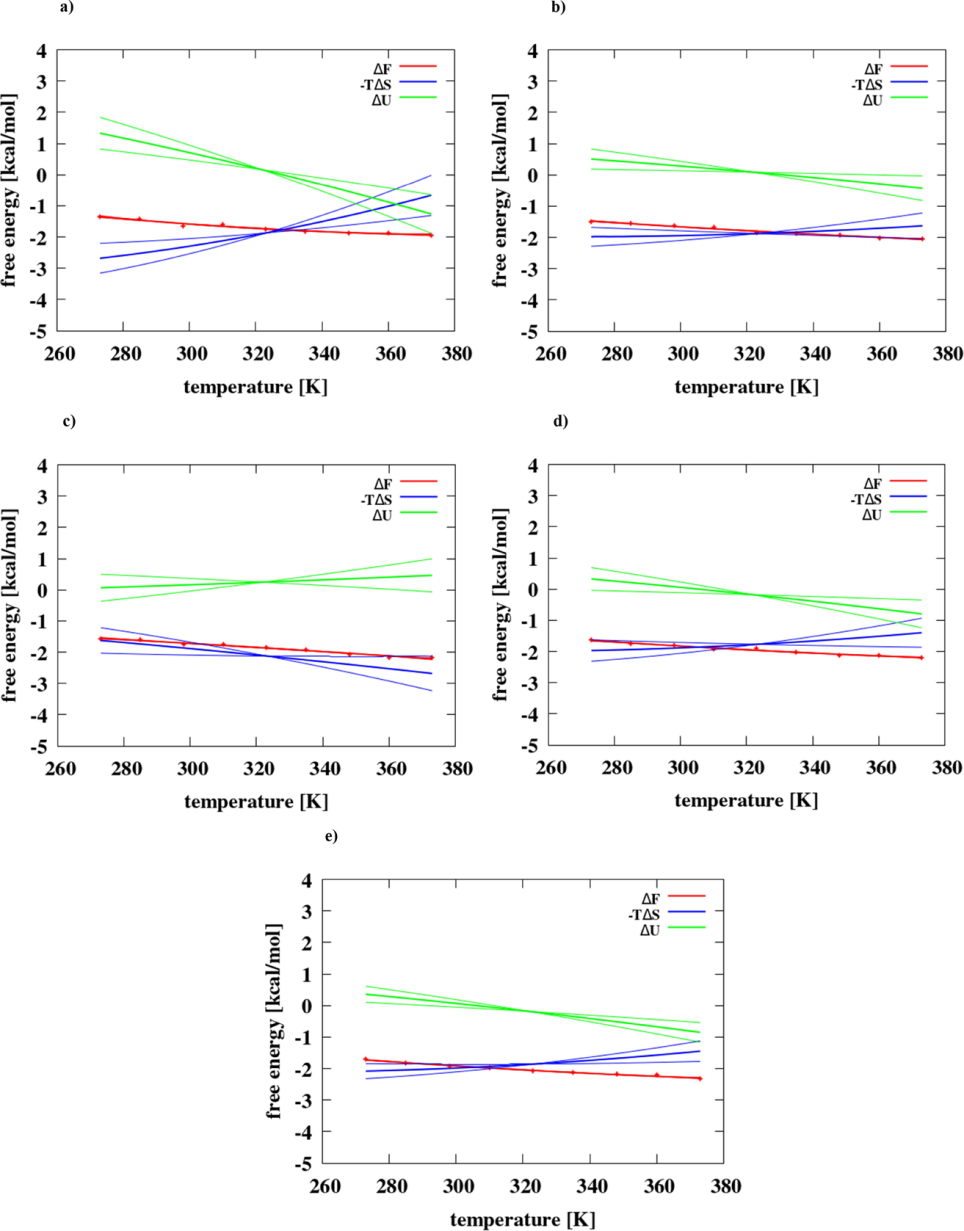
Changes of the free energy, energy, and entropy associated with the formation of the contact minimum for the adamantane dimer as a function of temperature in (a) IS = 0 mol/dm^3^, (b) IS = 0.40 mol/dm^3^, (c) IS = 1 mol/dm^3^, (d) IS = 1.5 mol/dm^3^, and (e) IS = 2 mol/dm^3^. Thin lines show errors for enthalpy and entropy fitting.

**Figure 4:**
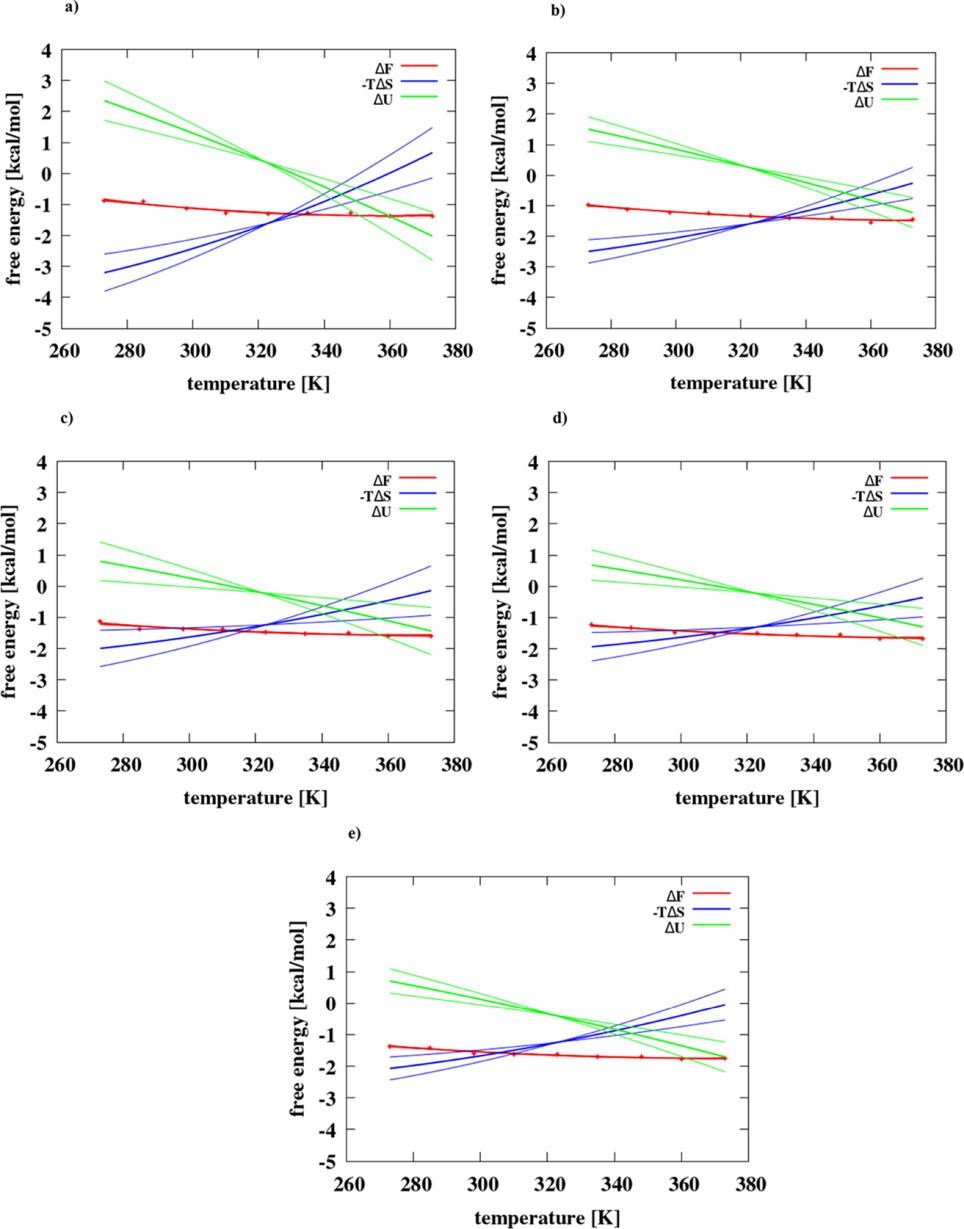
Changes of the free energy, energy, and entropy associated with the formation of the contact minimum for the hexane dimer as a function of temperature in (a) IS = 0 mol/dm^3^, (b) IS = 0.40 mol/dm^3^, (c) IS = 1 mol/dm^3^, (d) IS = 1.5 mol/dm^3^, and (e) IS = 2 mol/dm^3^. Thin lines show errors for enthalpy and entropy fitting.

As can be seen from Figures 1 and 2, the depth of the CM increases as the temperature increases, a feature which has also been observed by other authors.^5,12,13^ It can, therefore, be concluded that the tendency to the hydrophobic association and, consequently, the strength of hydrophobic interactions of the molecules studied increases with temperature. The dependence of Δ*F*(CM) on temperature is plotted as shown in Figures 3 and 4. Moreover, the position of the DM is shifted toward greater distances at each value of ionic strength. In most cases, these maxima are also getting lower when temperature increases, i.e., the desolvation energy barrier is getting smaller. It can also be seen from the graphs that, generally, the depth of the CM increases with ionic strength, which is consistent with the results obtained by other researchers.^14^

### Thermodynamics of Hydrophobic Association

**Figure 5:**
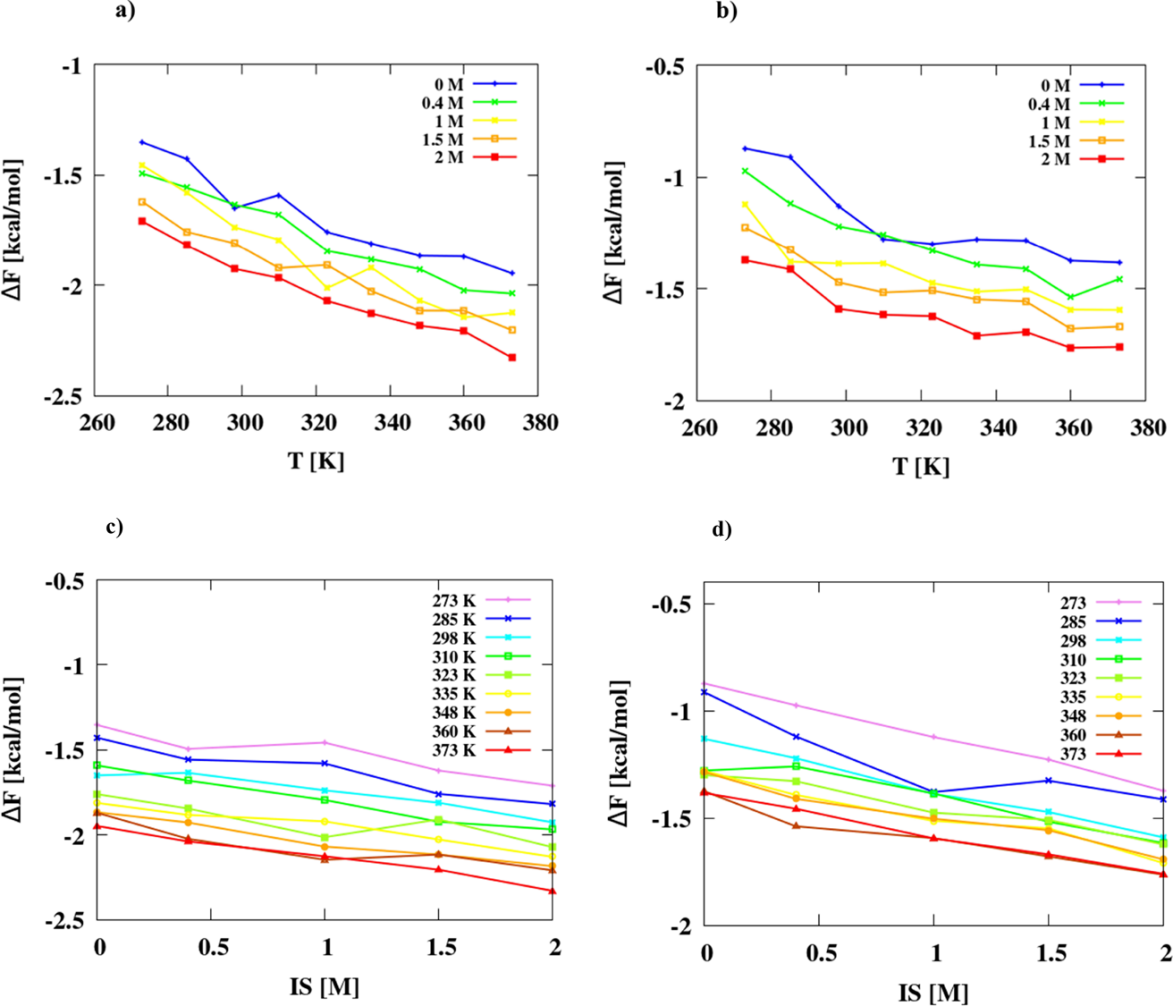
Plots of the dependence of the PMF (free energy) at the contact minimum on temperature (a, b) and ionic strength (c d) for adamantane [figures (a) and (c)] and hexane [figures (b) and (d)] dimers, respectively.

The plots of Δ*F*(CM) as functions of temperature are shown in Figure 5a,b and those of ionic strength are shown in Figure 5c,d for adamantane (Figure 5a,c) and hexane (Figure 5b,d), respectively. As stated in the preceding section, Δ*F*(CM) decreases with both temperature and ionic strength. The dependence of Δ*F*(CM) on ionic strength is effectively linear, while that on temperature exhibits a positive curvature, which is greater for hexane (Figure 5b) suggesting a substantial negative heat capacity change upon the hydrophobic association, as detailed below.

To perform a more detailed analysis of the thermodynamics of hydrophobic association, for each ionic strength value, we determined the changes of excess energy (Δ*U*) and excess entropy (−*T*Δ*S*) contributions to the PMF at the CM as a function of temperature and ionic strength (eqs 3 and 4). The respective plots are shown in Figures 3 and 4.

It can be seen from Figures 3 and 4 that except for the adamantane dimer at IS = 1 M the Δ*U* increases and −*T*Δ*S* decreases with temperature. For the adamantane dimer at IS = 1 M (Figure 3c), the average Δ*U*(*T*) increases with temperature. The slopes of the temperature dependence of Δ*U* and −*T*Δ*S* decrease with increasing salt concentration and are also greater for hexane (Figure 4) compared to those of the adamantane dimer (Figure 3).

**Figure 6:**
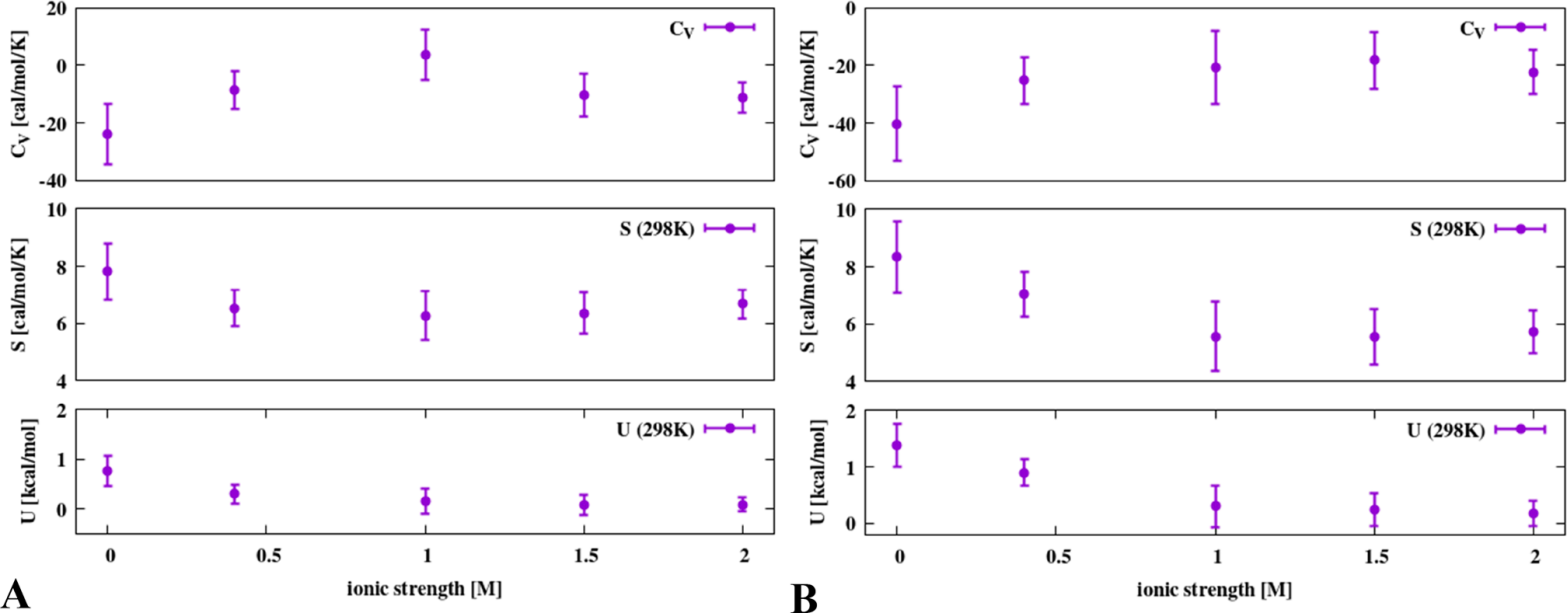
Plots of the changes in heat capacity, entropy at *T* = 298 K, and energy at *T* = 298 K following the formation of adamantane (A) and hexane (B) dimers at the contact minimum with the ionic strength.

To get a better insight into the thermodynamics of CM formation, we analyzed the dependence of excess heat capacity [Δ*C*_V_(CM)], excess entropy [Δ*S*(CM)], and excess energy [Δ*U*(CM)] at the contact minimum on ionic strengths for all four simulation temperatures at *T* = 298 K. The plots are shown in Figure 6A,B for the adamantane and hexane dimer, respectively. It can be seen from the figure that, consistent with the results of previous studies,^25,26^ Δ*C*_V_(CM) is negative except for adamantane at IS = 1 M; however, the error bar of this point extends toward negative values (the top panel of Figure 6A). Δ*C*_V_(CM) is the most negative for IS = 0 M and generally asymptotically increases (becomes less negative) with increasing ionic strength. The excess heat capacity is about twice as negative for the hexane dimer (about −40 cal mol^–1^ K^–1^ at IS = 0 M and about −20 cal mol^–1^ K^–1^ at IS = 2 M) as compared to the adamantane dimer (about −20 cal mol^–1^ K^–1^ at IS = 0 M and about −10 cal mol^–1^ K^–1^ at IS = 2 M). Δ*U*(CM) and Δ*S*(CM) are positive and decrease with increasing the ionic strength. The negative sign of Δ*C*_V_(CM) corresponding to the hydrophobic association is explained in terms of the increase of heat capacity upon hydrophobic hydration, which is due to the organization of water molecules around the hydrophobic particle.^27^ Consequently, the formation of a hydrophobic dimer, which removes some of the water molecules from the hydration sphere, results in a negative excess heat capacity. The decrease of excess heat capacity with increasing ionic strength suggests that the difference between water organization around a hydrophobic particle and the bulk of the solvent becomes less pronounced because the introduced ions also contribute to water organization.

**Figure 7:**
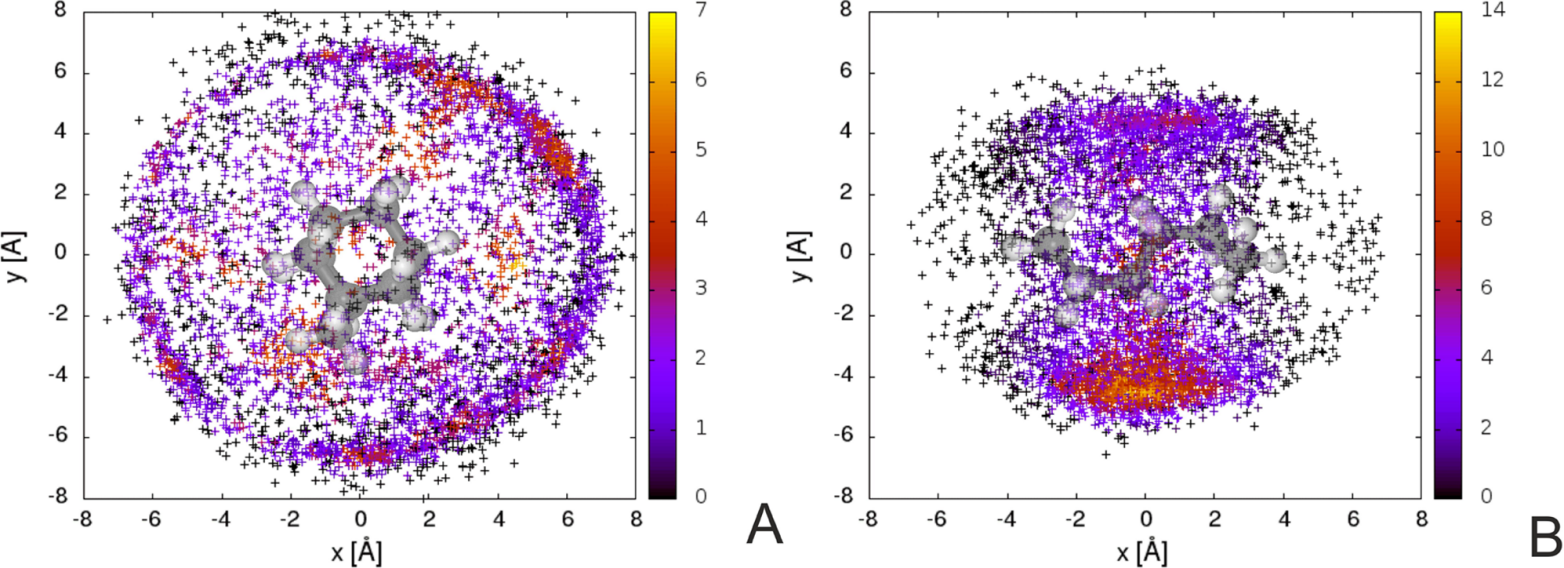
Scatter plots of the distributions of the center of the mass of the second hydrophobe molecule concerning that of the first one for adamantane (A) and hexane (B) dimers near the contact minimum. The reference system is centered at the center of the mass of the first molecule. For the adamantane molecule, the *x*-axis runs from the mass center to one of the carbon atoms bonded to three other carbon atoms (C_1_), while the y-axis is in the plane defined by the C_1_, and one of the carbon atoms bonded to C_1_ (C_2_). For the hexane molecule, which has a prolate shape, the horizontal (*x*) axis is the long axis of the molecule and the vertical (*y*) axis is the second-longest axis. For clarity, only every 10th snapshot has been taken. The points are colored according to the number of the neighboring points divided by 10, the color scale shown is in the right panel. The molecules (hexane in one of the possible conformations) are superposed on the panels.

The difference in the values of the excess heat capacity between the adamantane and hexane dimer must result from the prolate shape of the hexane molecule, which can be expected to result in different behaviors of solvation-shell water. The fact that the solvation pattern is different is evident from Figure 7 A,B in which the positions of the center of the mass of the second hydrophobe molecule in the coordinate system of the first molecule, specified in the legend to Figure 7, are plotted for the configuration close to the contact minimum. As shown in Figure 7A, the center of the mass of the second adamantane molecule is distributed nearly spherically in the coordinate system of the first one, with higher concentrations corresponding to orientations where the second molecule can fit into the symmetrically distributed cavities in the molecular surface of the first one. Conversely, the center of the mass of the second hexane molecule is distributed to reflect the prolate shape of hexane and its distribution is concentrated about the shorter axis, this indicates the dominant side-to-side mutual orientation of the molecules as observed previously.^28^ This qualitative picture does not change with temperature or with salt concentration.

## Conclusions

In this work, we carried out a simulation study of the dependence of hydrophobic homodimer formation of two hydrocarbon molecules with different shapes: adamantane, which is a large rigid hydrophobic particle with nearly spherical symmetry, and hexane, which has a prolate shape. We determined the respective PMF profiles, as functions of CM distances, for a range of temperatures and ionic strengths. The PMF plots for the homodimer of the nearly spherical adamantane had a characteristic shape for hydrophobic interactions with the CM and the SSM separated by the DM. For hexane, the PMF plots had different shapes, regardless of temperature and ionic strength. The depth of the CM and, consequently, the strength of hydrophobic interactions were found to increase with increasing temperature, regardless of salt concentration. For the adamantane dimer, the depth of the CM steadily increases with increasing salt concentration while, for the hexane dimer, the CM is deeper for a concentration of 0.4 mol/dm^3^ compared to pure water but then the depth does not change much with increasing salt concentration.

An analysis of the excess thermodynamic quantities of the hydrophobic association of the two hydrocarbon molecules studied corresponding to the CM enables us to conclude that the presence of salt most apparently influences the excess heat capacity. For both hydrophobic dimers, the excess heat capacity is negative and asymptotically decreases in absolute value with increasing salt concentration. This behavior can be interpreted in terms of salt-induced ordering of the water molecules in the hydration sphere, which leads to increased heat capacity.^27^ Hydrophobic dimer formation, which results in reducing the solvation sphere and, consequently, decreasing the number of water molecules in it, causes the excess heat capacity to decrease. The excess heat capacity is more negative for hexane (from about −40 cal mol^–1^ K^–1^ for pure water to about −20 cal mol^–1^ K^–1^ for IS = 2 mol/dm^3^). This difference could probably result from the prolate shape of the hexane molecule compared to the largely rigid and nearly spherical adamantane molecule. This, in turn, suggests that the studies of hydrophobic hydration and association in which rigid and nearly spherical hydrophobic particles are used are probably not sufficient to conclude the nature of the hydrophobic phenomena that occur in soft matters, especially in biological systems (e.g., the formation of lipid membranes or in protein folding) in which salts are a necessary constituent.
